# Brain death induces renal expression of heme oxygenase-1 and heat shock protein 70

**DOI:** 10.1186/1479-5876-11-22

**Published:** 2013-01-29

**Authors:** Leon FA van Dullemen, Eelke M Bos, Theo A Schuurs, Harm H Kampinga, Rutger J Ploeg, Harry van Goor, Henri GD Leuvenink

**Affiliations:** 1 Departments of Surgery, University Medical Center Groningen, University of Groningen, Hanzeplein 1, PO Box 30.001, 9700 RB, Groningen, The Netherlands; 2 Pathology and Laboratory Medicine, University Medical Center Groningen, University of Groningen, Hanzeplein 1, PO Box 30.001, 9700 RB, Groningen, The Netherlands; 3 Radiation and Stress Cell Biology, University Medical Center Groningen, University of Groningen, Hanzeplein 1, PO Box 30.001, 9700 RB, Groningen, The Netherlands

**Keywords:** Kidney, Protective genes, Rat, Organ donation, HSP, HSP70, HSP40, HSP27

## Abstract

**Background:**

Kidneys derived from brain dead donors have lower graft survival and higher graft-function loss compared to their living donor counterpart. Heat Shock Proteins (HSP) are a large family of stress proteins involved in maintaining cell homeostasis. We studied the role of stress-inducible genes Heme Oxygenase-1 (HO-1), HSP27, HSP40, and HSP70 in the kidney following a 4 hour period of brain death.

**Methods:**

Brain death was induced in rats (n=6) by inflating a balloon catheter in the epidural space. Kidneys were analysed for HSPs using RT-PCR, Western blotting, and immunohistochemistry.

**Results:**

RT-PCR data showed a significant increase in gene expression for HO-1 and HSP70 in kidneys of brain dead rats. Western blotting revealed a massive increase in HO-1 protein in brain dead rat kidneys. Immunohistochemistry confirmed these findings, showing extensive HO-1 protein expression in the renal cortical tubules of brain dead rats. HSP70 protein was predominantly increased in renal distal tubules of brain dead rats treated for hypotension.

**Conclusion:**

Renal stress caused by brain death induces expression of the cytoprotective genes HO-1 and HSP70, but not of HSP27 and HSP40. The upregulation of these cytoprotective genes indicate that renal damage occurs during brain death, and could be part of a protective or recuperative mechanism induced by brain death-associated stress.

## Background

Renal transplantation leads to a significantly improved prognosis for the majority of patients with end-stage renal disease. Most kidneys used for renal transplantation are derived from brain dead donors. These kidneys, however, are known to have inferior organ quality and survival compared to kidneys from living related donors [1,2]. The knowledge on the (patho)-physiological processes initiated by brain death that are responsible for this observation are not fully elucidated. Previous studies indicated that the brain death causes marked inflammatory changes in peripheral organs [3] and incites various (patho)-physiological processes, like hemodynamic instability [4] and hormone dysregulation [5]. The inflammatory changes in liver and kidney, as evidenced by an increase in CD45^+^ leukocytes, coincide with upregulation of cell adhesion molecule expression (ICAM-1, VCAM-1, E-selectin) and apoptosis [6-9]. The hemodynamic instability, displayed by a short hypertensive phase at the onset of brain death followed by a decline in blood pressure to normal tension or hypotension, could cause poor organ perfusion and thereby an inadequate oxygen delivery to the peripheral tissues, resulting in organ damage.

Heat-shock proteins (HSP) are a diverse group of proteins involved in cellular homeostasis and display cytoprotective effects in different forms of stress, mostly ascribed to their role as molecular chaperones. HSPs act in, for example, protein folding, protein assembly, protein aggregation, intracellular transport, and degradation of damaged substances (for review, see references [10,11]). It has been shown that heat-shock proteins, which are numbered according to their approximate molecular mass in kDa, have a protective role in ischemia/reperfusion injury by repairing damaged proteins and thereby removing the stimulus for apoptosis [10,12-20]. Upregulation of HSP70 prior to transplantation improves graft survival and function [19-22]. Selective upregulation of heme oxygenase-1 (HO-1, also known as HSP32) has shown similar graft-protective effects in the kidney, heart and liver [23-27]. Furthermore, HO-1 has immunosuppressive characteristics [25,28,29], that are likely mediated in part by production of carbon monoxide (CO) during heme degradation, which possibly induces vasodilatation and attenuates pro-inflammatory processes during brain death.

There is only very limited knowledge about the effects of brain death on the expression of HSPs in the kidney. To evaluate possible renal recuperative and/or protective mechanisms induced during brain death, we studied kidneys from rats exposed to a 4 hour period of brain death for presence of HSP27, HSP40, HSP70, and HO-1 protein and mRNA.

## Methods

### Animals

Adult male Fisher F344 rats weighing 278–310 grams were used (Harlan, Horst, The Netherlands) in this study. Average weight was 285±18 grams. The rats were housed in a light- and temperature-controlled environment and had free access to food and water. All animals received care in compliance with the guidelines of the local animal ethics committee according to Experiments on Animals Act (1996) issued by the Ministry of Public Health, Welfare and Sports of the Netherlands.

### Brain death induction

Rats were anesthetized using isoflurane with a mixture of NO_2_ and O_2_ during brain death induction. They were intubated via a tracheostomy by a blunt-tipped cannula and mechanically respirated. The animals were ventilated on a small rodent ventilator (Zoovent, Triumph Technical Services Ltd, UK) with 4 liter/min O_2_ in air for 30 min (stroke rate: 46 per min, inspiration/expiration: 1/2, peak inspiratory pressure: 16 mmHg, mean inspiratory pressure: 6 mmHg, and a positive end expiratory pressure: 0 mmHg). After that, they were ventilated with 0.5 liter/min O_2_ in air until the end of the brain death period.

Brain death induction was performed by drilling a 1×1 mm hole through the frontomedial part of the skull, just lateral of the central vein. A no.4 Fogarty catheter (Edwards Life Sciences Co., Irvine, CA, USA) was inserted in the epidural space with the tip pointing caudally. To simulate an epidural haematoma leading to brain death, intracranial pressure was increased gradually by slow inflation of the balloon (16 μl saline per min) using a syringe pump (Terufusion, Termo Co., Tokyo, Japan). Inflation of the balloon was stopped after approximately 25 min, when the blood pressure reached normal levels after an initial phase of hypotension. During balloon inflation, the sharp rise of blood pressure that is typical in brain death development was seen. Brain death was verified 30 minutes after the start of balloon inflation by an apnoea test, confirmation of dilated and fixed pupils and the absence of corneal reflexes. The average balloon volume that consistently induced brain death was (420±20 μl). The balloon was kept inflated during the entire experiment. Blood pressure was monitored via a PE cannula (0.40×0.80 mm) implanted in the left femoral artery. Blood pressure above 80 mmHg was considered normotensive. When blood pressure dropped beneath 80 mmHg, rats were infused with 10% hydroxyethyl starch (HAES) until normotension was reached. Body temperature, measured with a rectal thermoprobe (Mon-a-therm 6510, Mallinckrodt Medical, Inc., St. Louis, MO, USA), was maintained at 37°C by means of a heating pad and lamp.

After 4 hours of brain death, isoflurane 0.5% anaesthesia with a mixture of NO_2_ and O_2_ was used 10 minutes before the end of the brain death period to achieve full muscle relaxation in order to allow abdominal surgery. Left kidneys were removed after perfusion with cold saline and cut in two sections, one part was snap frozen in liquid nitrogen, the other part was fixated in 4% paraformaldehyde.

### RNA isolation and semi quantitative RT-PCR

Total RNA was isolated from snap frozen renal tissue using the SV Total RNA Isolation System (Promega, Madison, Wisconsin, USA) according to the manufacturer’s protocol. Briefly, cDNA synthesis from total RNA included an initial step in which 1 μg of RNA is incubated with T_11_VN oligo’s (0.5 μg) for 10 minutes at 70°C. Subsequently, buffer, dNTP’s (final conc. 1 mM), DTT (final conc. 10 mM), 200 U M-MLV Reverse Transcriptase and 20 U RNaseOUT™ Recombinant Ribonuclease Inhibitor (all from Invitrogen, Carlsbad, California, USA) were added and incubated for 50 min at 37°C, after which the reaction was inactivated by a 15 min incubation at 70°C. Two μl of cDNA was amplified by PCR in a buffer consisting of 0.2 mM dNTP’s, 1.5 mM MgCl_2_, 1x PCR buffer and 1 U Taq DNA polymerase (all from Invitrogen). Gene-specific primer pairs (0.5 μM each) were added directly, after which they were heated for 3 min at 94°C. PCR was performed on a T1 Thermocycler (Biometra) and cycles consisted of 94°C for 40 s, a primer annealing temperature at 56°C for 40 s and a polymerization step at 72°C for 40 s. The numbers of cycles were selected to allow amplification within the linear range.

The primer sequences, the number of cycles and their respective PCR fragment lengths were:

HO-1: 5’-ACTTTCAGAAGGGTCAGGTGTCC-3’, 5’-TTGAGCAGGAAGGCGGTCTTAG-3’,

28 cycles, 523 bp;

HSP27: 5’-AAGGCTTCTACTTGGCTCCAG-3’, 5’-ACATGGCTACATCTCTCGGTG-3’,

26 cycles, 226 bp;

HSP40, 5’-GAAACTGCAACCACAGAGAGC-3’, 5’-GGATGGAAATCCCTGACTAGC-3’,

40 cycles, 275 bp;

HSP70, 5’-CTGACAAGAAGAAGGTGCTGG-3’, 5’-AGCAGCCATCAAGAGTCTGTC-3’,

25 cycles, 302 bp.

Primer pairs were developed using Primer3 (http://bioinformatics.weizmann.ac.il/cgi-bin/primer/primer3.cgi). Ethidium bromide-stained agarose gels were scanned on Image Master® VDS (Amersham Biosciences, UK) using LISCAP software. PCR product abundance was quantified using Imagemaster 1D Elite (Amersham) and normalized for the abundance of the β-actin signal from the same cDNA.

### Western blotting

Per sample, three 20 μm cryostat sections were lysed in 200 μL RIPA buffer (1% NP_40_, 0.1% SDS, 10 mM β-mercaptoethanol) containing protease inhibitors (Complete, Roche). Samples were lysed on ice, centrifuged for 15 min at 16000g (4°C) and supernatants collected. Protein concentrations were measured using the Lowry Protein assay (BioRad). 7.5 μg of total protein was separated by SDS-PAGE (Sodium Dodecyl Sulphate PolyAcrylamide Gel Electrophoresis) on 10% polyacrylamide gels using the BioRad electrophoresis system. Proteins were electroblotted (BioRad blotting system) onto PVDF membranes (Hybond, Amersham) after which immune detection was performed. Briefly, membranes were blocked in PBS (6.5 mM Na_2_HPO_4_, 1.5 mM KH_2_PO_4_, 2.7 mM KCl and 150 mM NaCl; pH 7.2) with 0.05% Tween (PBST) containing 5% SKIM, overnight. Incubation with primary antibodies directed to HSP27 (SPA-801), HSP40 (SPA-400), HSP70 (SPA-810) and HO-1 (OSA-111, all by StressGen, Victoria, BC, Canada) for 2 hours was followed by 1 hr incubation with an appropriate secondary antibody conjugated to peroxidase in blocking buffer at 1:2000 dilution. After each incubation step, membranes were washed 5 times in PBST. Bound antibodies were detected with ECL Western blotting detection system (Amersham, UK) according to the manufacturer’s protocols. Detected signal was quantified and normalized for the GAPDH signal on the same blot.

### Immunohistochemistry

For localization of HSP40, HSP70 and HO-1, paraffin sections (4 μm) were de-waxed, rehydrated and subjected to heat-induced antigen retrieval by microwave heating in 10 mM citrate buffer (pH=6.0, HSP40), 1 mM EDTA (pH=8.0, HSP70), or by overnight incubation in 0.1 M Tris/HCl buffer at 80°C (pH=9.0, HO-1). Endogenous peroxidase was blocked with 0.03% H_2_O_2_ in phosphate-buffered saline (PBS) for 30 minutes. Primary antibodies used were identical to those used in Western blotting. Incubation lasted for 60 min at RT and binding of the antibody was detected by sequential incubation with appropriate peroxidase-labelled secondary and tertiary antibodies (Dakopatts, Glostrup, Denmark) for 30 min. Antibody dilutions were made in phosphate-buffered saline (PBS) supplemented with 1% bovine serum albumin (BSA) and 1% normal rat serum. The peroxidase activity was visualised using 3,3’-diaminobenzidine tetrahydrochloride (DAB+, cat. no. K3468; DAKO). Finally, sections were counterstained with haematoxylin.

For HSP27 staining, cryostat sections (4 μm) were fixed in acetone for 10 min. Subsequently, they were dried and incubated with the primary antibody (SPA-801, StressGen) for 60 min at RT. Endogenous peroxidase was blocked with 0.0075% H_2_O_2_ in PBS for 30 min. Detection of binding was performed as described above. Peroxidase activity was visualised using 3-amino-9-ethylcarbazole (AEC) for 15 min. Sections were counterstained with haematoxylin.

### Statistical analysis

Statistical analysis was performed using the two-sided Students T test, a *p* value <0.05 was considered significant. Correlations between variables were assessed with one-way ANOVA. Statistical analyses were performed using SPSS version 18.0 (SPSS Inc, Chicago, US).

## Results

### Semi-quantitative RT-PCR

**Figure 1:**
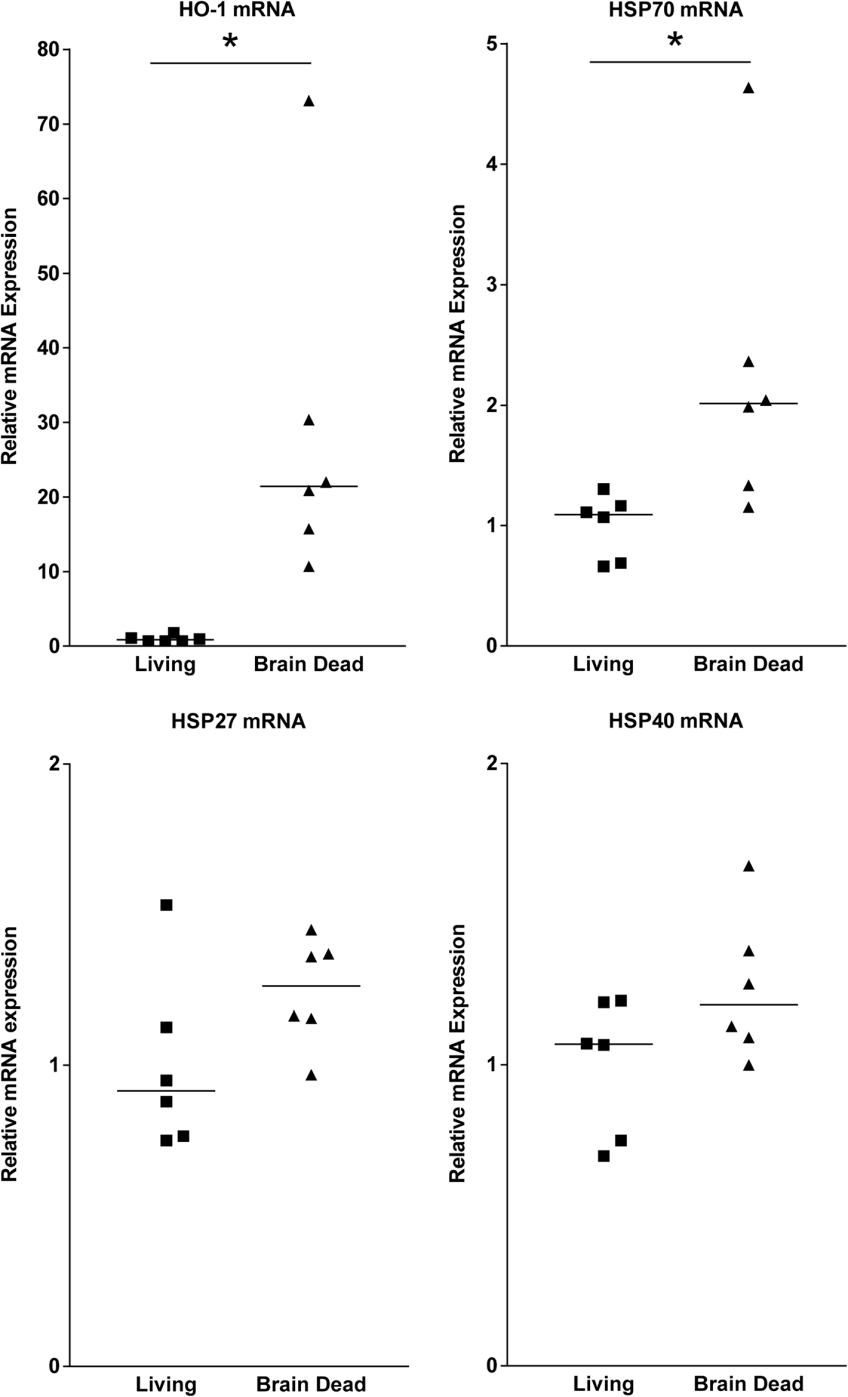
**qPCR results showed a massive increase in renal HO-1 mRNA levels of brain dead rats.** A significant increase in HSP70 expression was also seen. qPCR for HSP27 and HSP40 did not reveal differences in mRNA expression between control and brain dead groups.

Renal expression of genes coding for HO-1 and HSP70 was significantly increased in kidneys of brain dead rats compared to living controls (Figure 1). For HO-1 the increase was 3.7 fold compared to controls (p<0.05), HSP70 was increased 2.4 fold (p<0.05). Differences in the expression of HSP27 and HSP40 were not observed (Figure 1). Rats treated with substantial volumes of hydroxyethyl starch (1.0-5.75 mL, n=3) for blood pressure regulation showed a 3.8 fold (p<0.05) increased expression of HSP70 compared to control rats. Rats treated with scant volumes of hydroxyethyl starch (0–0.6 mL, n=3) showed a 1.7 fold increase, which was not significant (p=0.25).

### Western blotting

**Figure 2:**
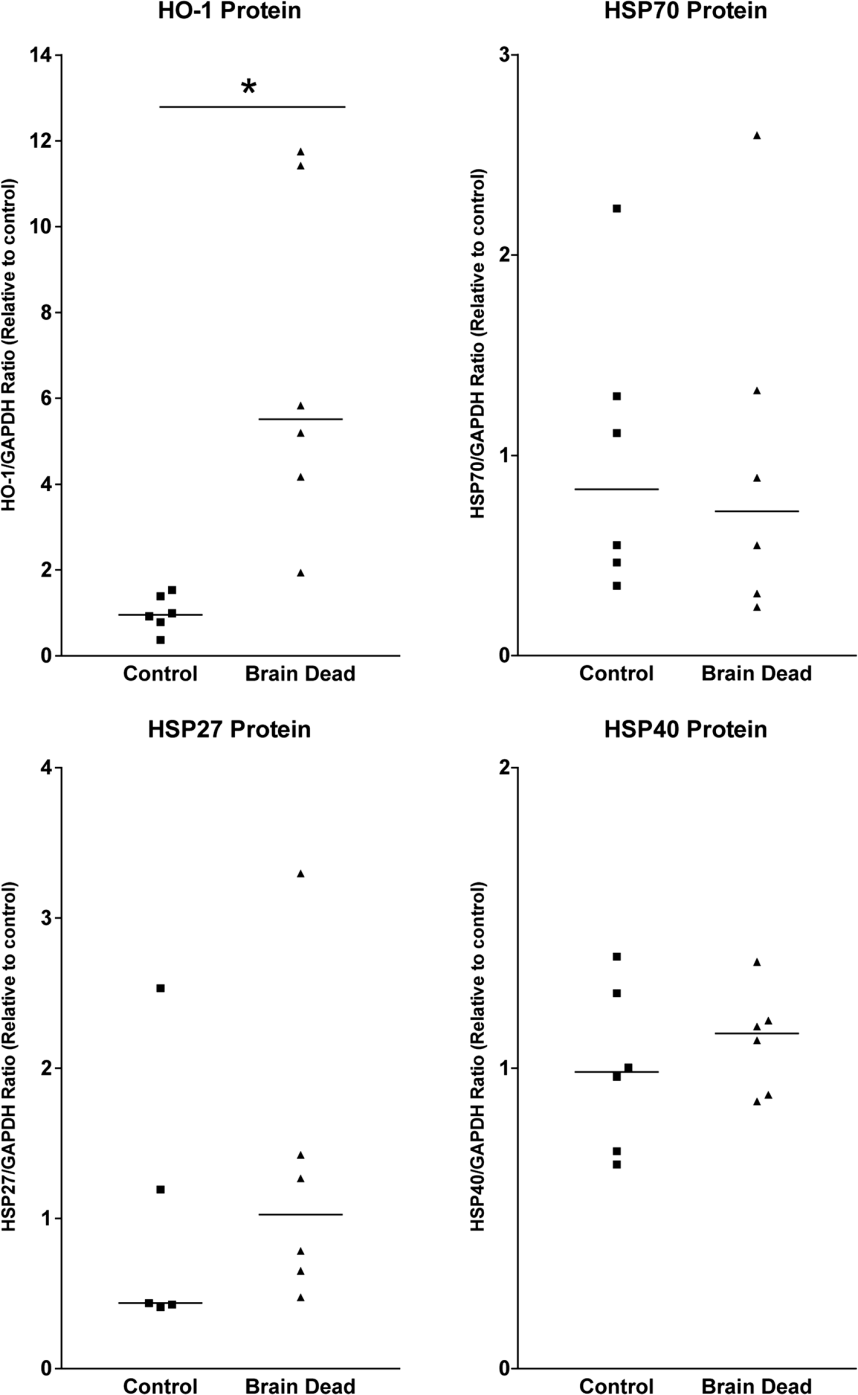
**Western blotting showed a great increase in HO-1 protein levels.** However, the increase in HSP70 mRNA did not lead to measurable differences in protein levels at this time point. Protein levels of HSP27 and HSP40 did not differ between control and brain dead groups.

**Figure 3:**
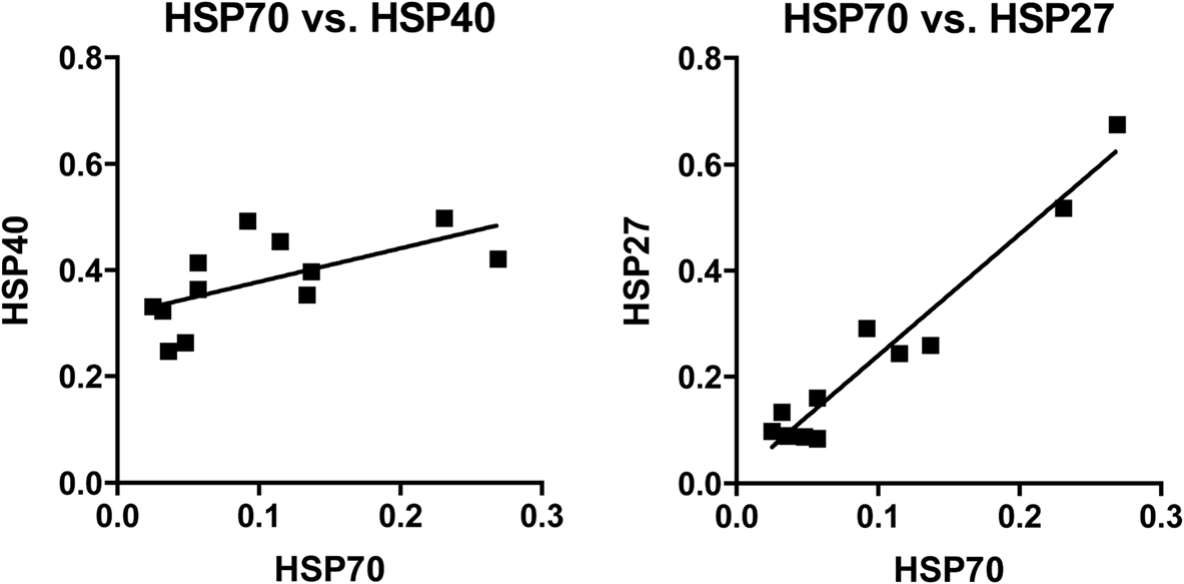
**Renal Western blot results from living and brain dead rats for HSP70 protein expression was found to correlate significantly (R**^**2**^**=0.38) with HSP40 protein expression.** HSP70 protein expression was also found to correlate significantly (R^2^=0.94) with HSP27 protein expression.

A 4.6 fold increase (p<0.001) in HO-1 protein was observed in brain dead rat kidneys compared to living controls (Figure 2). Quantities of HSP70 protein did not differ in the brain dead group (Figure 2), and no differences were detected between the normotensive and hypotensive groups of brain dead rats. Levels of HSP27 and HSP40 protein showed no variation between brain dead and control groups (Figure 2), confirming data from immunohistochemistry. Furthermore, we found significant positive correlations between the protein expression of HSP70 and the protein expressions of HSP40 (p<0.05, R^2^=0.38) and HSP27 (p<0.05, R^2^=0.94) (Figure 3).

### Immunohistochemistry

**Figure 4:**
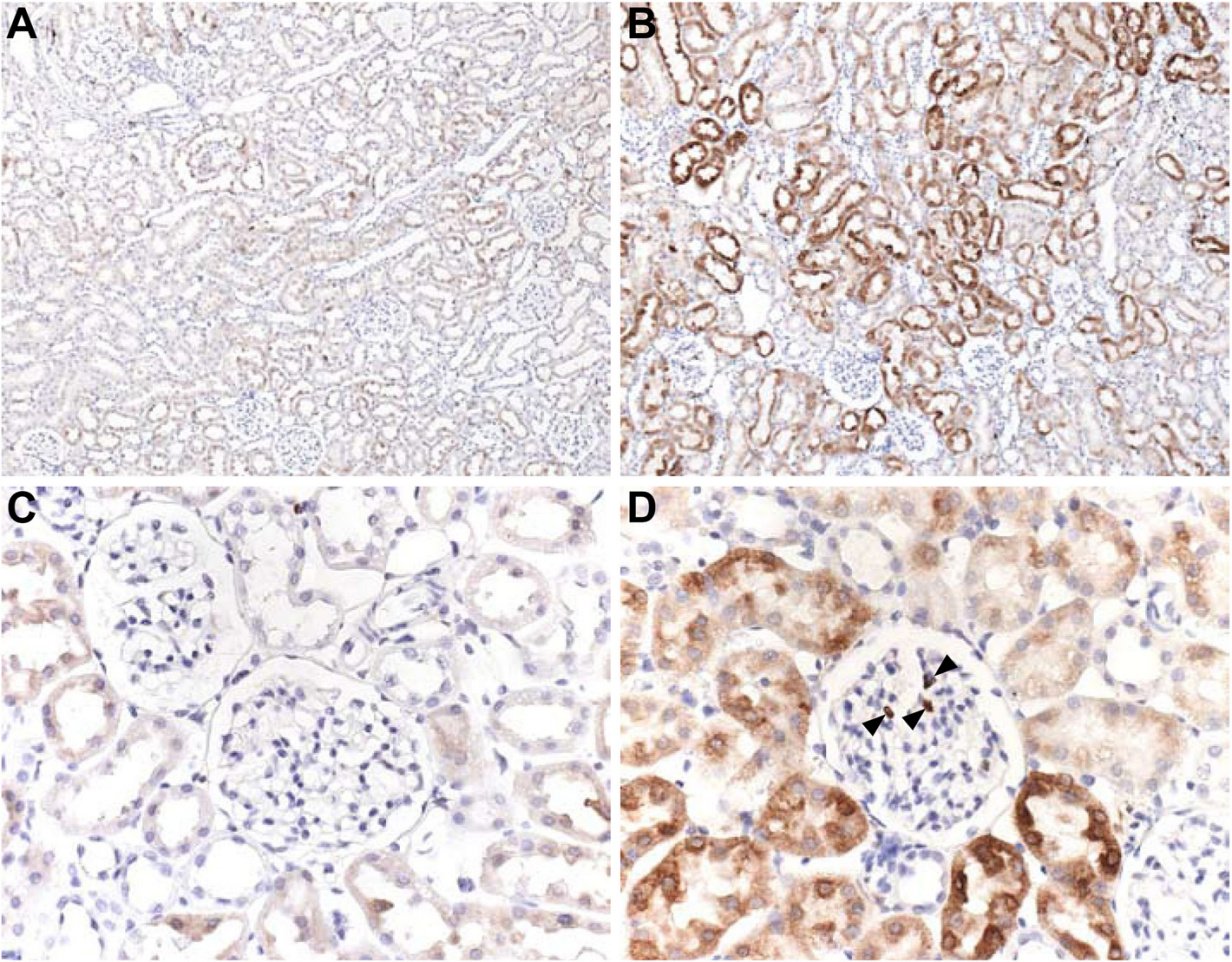
**HO-1 immunohistochemistry staining on renal tissue from living (A,C) and brain dead rats (B,D) showed a significant increase in HO-1 protein in renal cortical tubules of the 4 h brain dead group (B, 100x) compared to the living group (A 100x).** At a magnification of 400x (**C**,**D**) it is apparent that there is an increase in HO-1 protein in a number of singular tubular cells of brain dead rats. Also, several positive cells can be observed in the glomerulus (**D**, Arrowheads) which could be CD68^+^ macrophages.

In control rats, weak cytoplasmatic HO-1 staining of some proximal tubules of the renal cortex was observed (Figure 4A). Glomeruli and arteries were negative, as well as the collecting ducts and loops of Henle. In brain dead rat kidneys at 4 hours after induction, HO-1 was massively upregulated in the cortical proximal tubules (Figure 4B). Also, some solitary tubular cells showed very intense staining compared to adjacent cells (Figure 4D), which was not seen in controls (Figure 4C). Glomeruli were negative, with the exception of some solitary cells, which could be CD68-positive macrophages.

**Figure 5:**
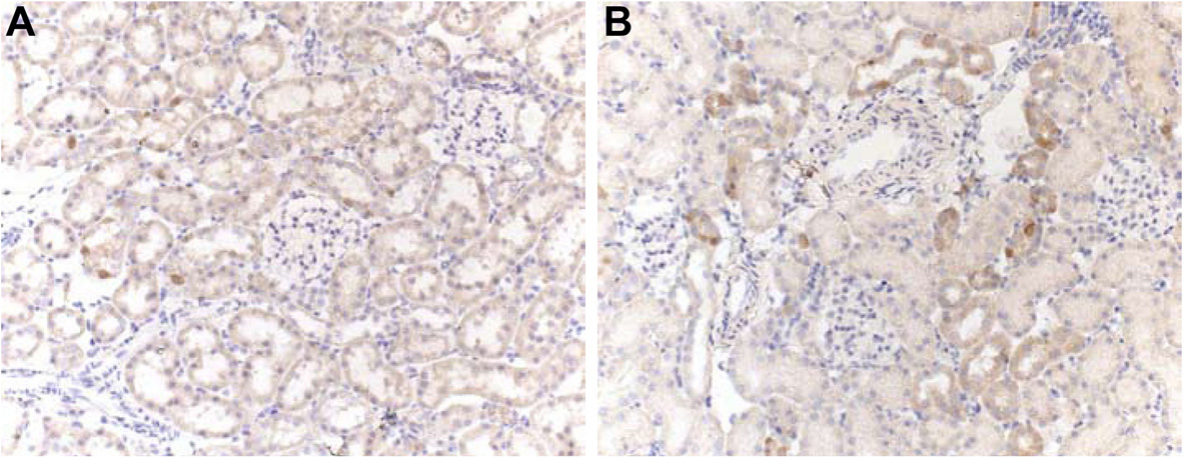
**HSP70 immunohistochemistry staining on renal tissue from living (A) and brain dead rats (B) at 100x magnification shows a slight increase in staining in the renal cortical tubules of the brain dead rats.** (**B**) Control kidneys showed only occasional positively stained singular cells in tubular structures.

HSP70 staining was found in some single distal tubular cells. Glomeruli were stained negative for HSP70 (Figure 5A). Upregulation of HSP70 was predominantly seen in the renal distal tubules of the hypotensive rats treated with hydroxyethyl starch as a volume replacement, showing an increase in the number of positively stained single cells (Figure 5B). In rats that remained normotensive during brain death and thus were not treated with hydroxyethyl starch, no difference in staining of HSP70 compared to controls was detected.

HSP27 was present in vascular smooth muscle cells of renal arteries. Tubular staining was not detected, but some glomerular staining was evident.

HSP40 was detected in the glomeruli, with visceral and parietal epithelium stained positive. Also, some distal tubular and collecting duct staining was detected.

No detectable differences were found for HSP27 and HSP40 between the brain dead and control group, confirming Western blot results.

## Discussion

The major finding of the present study is that systemic stress associated with brain death induces enhanced expression of protective genes in the donor kidney. These data are in line with previous micro-array data showing marked upregulation of various protective genes during experimental brain death [30]. Since HO-1 is known to attenuate damage in various stress models, such as ischemia/reperfusion and experimental transplantation, we consider the significant increase of HO-1 protein and mRNA in renal proximal tubules from brain dead rats a recuperative mechanism in response to the injury caused by the systemic effects of extensive brain injury.

Brain death induction can cause significant detrimental effects on various donor organs. The “autonomic storm”, an immediate release of massive amounts of catecholamines into the blood stream, occurs directly after the onset of brain death. The hemodynamic changes cause altered perfusion of the kidneys, possibly resulting in ischemic damage [4,31]. As a result of brain death and its systemic effects increased pro-inflammatory reactivity in donor organs takes place, as evidenced by upregulation of cell adhesion molecules such as ICAM and selectins. The increments in adhesion molecules are associated with increased influx of inflammatory cells such as T-cells, macrophages and polymorphonuclear neutrophils. These early pro-inflammatory changes further progress to renal fibrotic damage, a decrease in renal function, and inferior graft survival [7,32,33].

Using immunohistochemistry in control rat kidneys, we noted weak HO-1 staining in the proximal tubules, indicating a physiological role for HO-1 in the kidney. Basal expression of HO-1 in the normal human kidney, although weak, is also found in the renal proximal tubules [34] and in some CD68^+^ macrophages as is described by Takano et al. [35]. In human renal disease, proximal tubular induction of HO-1 is found in patients with focal segmental glomerulosclerosis and rapidly progressing glomerulonephritis [34]. Also, massive renal upregulation of HO-1 mRNA is seen after ischemic acute renal failure in rats [36], as well as in a model of acute renal allograft rejection [37]. Although the mechanism of HO-1 induction was not studied here, these data point to defensive tubular adaptations that are induced in response to the induction of brain death.

In the normal rat kidney, HSP70 protein is found following a pattern resembling the corticopapillary concentration gradient, showing more HSP70 expression in the hyperosmotic medulla of the kidney compared to the isosmotic cortex [38]. Enhanced expression of HSP70 was also induced during brain death. However, the increased expression of HSP70 on protein level was small as observed by immunohistochemistry, and could not be reproduced by Western blotting. This could be the result of the relatively small amount of HSP70 inducing cells. The increase in HSP70 expression was predominantly noted in animals which were treated with hydroxyethyl starch for hemodynamic instability during brain death. Hydroxyethyl starch infusion can cause renal tubule lesions [39], is an independent risk factor for acute renal failure during severe sepsis [40] and has a negative effect on the outcome of renal transplantation if administered to brain dead patients before graft retrieval [39]. Therefore, we can not be sure if the induction of HSP70 is attributable to effects of brain death, brain death induced hemodynamic instability, the infusion of hydroxyethyl starch or any combination of these factors. However, in other studies, brain death induced HSP70 mRNA expression in the heart [41] and kidney [30].

In previous studies HSP27 in the human kidney showed a weak expression pattern in the cortical collecting ducts, and strong expression in the medullary collecting ducts. This pattern, similar to the expression pattern of HSP70, suggests the influence of the osmotic gradient on the expression of HSP27 [42]. HSP70, HSP40 and HSP27 are HSF-1-regulated proteins [43], and the correlations found between HSP70 and HSP40 protein expressions, and HSP70 and HSP27 protein expressions indicate that the increased HSP70 expression measured with RT-PCR and IHC is HSF-1 mediated. However, brain death induction did not have any effect on mRNA and protein expression of HSP40 or HSP27, these unchanged expression patterns of HSP40 and HSP27 suggest that these proteins are not upregulated fast enough to be involved in the recuperative cascade initiated during brain death.

The HSP family of proteins is upregulated in concert during heat-shock. We therefore investigated expression of multiple HSPs in our experimental brain death model. However, only HO-1 and HSP70 were found to be upregulated. The dissociation in induction of HSP in the various models suggests that different pathways are involved in the upregulation of these genes and depend on the initial stimulus. Yet, the effects of the increase in expression of HO-1 and HSP70 appear insufficient to counteract the detrimental effects of brain death to the donor kidney. The products of heme degradation probably mediate the protective effects of HO-1. Carbon monoxide, biliverdin and iron all have various cytoprotective effects in vitro and in vivo [44]. Part of the protection could be caused by CO mediated vasodilatation, anti-apoptosis or anti-inflammation. The other products of heme breakdown, iron and biliverdin, also have cytoprotective properties.

Pharmacological intervention boosting the expression of HO-1 and other HSPs prior to graft harvest could therefore be a valuable procedure to increase the performance of kidneys derived from brain dead donors. The immunoregulatory function of HO-1 has an important role in cardiac xenograft survival [45]. Induction of HO-1 during brain death could reduce the inflammatory processes initiated in the kidney. In fact, upregulation of HO-1 in brain dead rats using cobalt protoporphyrin (CoPP) in a model of experimental renal transplantation improved allograft survival to levels comparable to the living donor [46]. Interestingly, recent observations have shown that a longer duration of brain death influences kidney graft function and survival in a positive way [47]. Kidneys derived from donors that were subjected to a longer period of brain death showed a significant increase in graft survival, possibly reflecting a renal recuperative mechanism that may be mediated by the delayed induction of HSPs. Further study is needed to evaluate the role of protective and recuperative pathways during brain death, and their influence on renal transplant outcome.

## Conclusion

Renal stress caused by brain death induces expression of the cytoprotective genes HO-1 and HSP70, but not of HSP27 and HSP40. The up-regulation of these cytoprotective genes could be part of a recuperative mechanism induced by stress associated with brain death.

## Competing interests

The authors declare that they have no competing interests.

## Authors’ contributions

LD, EB, and TS carried out the animal experiments, the Western blotting, immunohistochemistry, and the RT-PCR. HG helped to evaluate the immunohistochemistry. HL and HK conceived the study, HL, HK, RP participated in its design. LD, EB, HK, HL helped to draft the manuscript. All authors read and approved the final manuscript.
